# Omega 3 fatty acid for the prevention of cognitive decline and dementia

**DOI:** 10.1590/S1516-31802012000600013

**Published:** 2013-01-18

**Authors:** Emma Sydenham, Alan D. Dangour, Wee-Shiong Lim

## Abstract

**BACKGROUND:**

Evidence from observational studies suggests that diets high in omega-3 long-chain polyunsaturated fatty acids (PUFA) may protect people from cognitive decline and dementia. The strength of this potential protective effect has recently been tested in randomized controlled trials.

**OBJECTIVES:**

To assess the effects of omega-3 PUFA supplementation for the prevention of dementia and cognitive decline in cognitively healthy older people.

**METHODS:**

*Search:* We searched ALOIS - the Cochrane Dementia and Cognitive Improvement Group’s Specialized Register on - 6 April 2012 using the terms: “omega 3”, PUFA, “fatty acids”, “fatty acid”, fish, linseed, eicosapentaenoic, docosahexaenoic.

*Selection criteria:* Randomised controlled trials of an omega-3 PUFA intervention which was provided for a minimum of six months to participants aged 60 years and over who were free from dementia or cognitive impairment at the beginning of the study. Two review authors independently assessed all trials.

*Data collection and analysis:* The review authors sought and extracted data on incident dementia, cognitive function, safety and adherence, either from published reports or by contacting the investigators for original data. Data were extracted by two review authors. We calculated mean difference (MD) or standardised mean differences (SMD) and 95% confidence intervals (CI) on an intention-to-treat basis, and summarized narratively information on safety and adherence.

**MAIN RESULTS:**

Information on cognitive function at the start of a study was available on 4080 participants randomised in three trials. Cognitive function data were available on 3536 participants at final follow-up. In two studies participants received gel capsules containing either omega-3 PUFA (the intervention) or olive or sunflower oil (placebo) for six or 24 months. In one study, participants received margarine spread for 40 months; the margarine for the intervention group contained omega-3 PUFA. Two studies had cognitive health as their primary outcome; one study of cardiovascular disease included cognitive health as an additional outcome. None of the studies examined the effect of omega-3 PUFA on incident dementia. In two studies involving 3221 participants there was no difference between the omega-3 and placebo group in mini-mental state examination score at final follow-up (following 24 or 40 months of intervention); MD-0.07 (95% CI -0.25 to 0.10). In two studies involving 1043 participants, other tests of cognitive function such as word learning, digit span and verbal fluency showed no beneficial effect of omega-3 PUFA supplementation. Participants in both the intervention and control groups experienced either small or no cognitive declines during the studies. The main reported side-effect of omega-3 PUFA supplementation was mild gastrointestinal problems. Overall, minor adverse events were reported by fewer than 15% of participants, and reports were balanced between intervention groups. Adherence to the intervention was on average over 90% among people who completed the trials. All three studies included in this review are of high methodological quality.

**AUTHORS’ CONCLUSIONS:**

Direct evidence on the effect of omega-3 PUFA on incident dementia is lacking. The available trials showed no benefit of omega-3 PUFA supplementation on cognitive function in cognitively healthy older people. Omega-3 PUFA supplementation is generally well tolerated with the most commonly reported side-effect being mild gastrointestinal problems. Further studies of longer duration are required. Longer-term studies may identify greater change in cognitive function in study participants which may enhance the ability to detect the possible effects of omega-3 PUFA supplementation in preventing cognitive decline in older people.

**This is the abstract of a Cochrane Review published in the Cochrane Database of Systematic Reviews (CDSR) 2012, issue 6, DOI:** 10.1002/14651858.CD005379.pub3 (http://onlinelibrary.wiley.com/doi/10.1002/14651858.CD005379.pub3/abstract;jsessionid=F0C2EAF3F22234CC89B1EF0F241F963A.d04t03). For full citation and authors details see reference1.

**The full text is freely available from:** http://www.cochranejournalclub.com/omega3-for-prevention-of-dementia-clinical/pdf/CD005379.pdf
